# Supplementary material for: Copper Ferrite Nanoparticles Synthesized Using Anion-Exchange Resin: Influence of Synthesis Parameters on the Cubic Phase Stability
Source: Materials (Basel). 2023 Mar 14;16(6):2318. doi: 10.3390/ma16062318 (PMC10059923; doi:10.3390/ma16062318)
Supplement: Supplementary file 1 [file materials-16-02318-s001.zip › materials-2276604-supplementary.pdf]

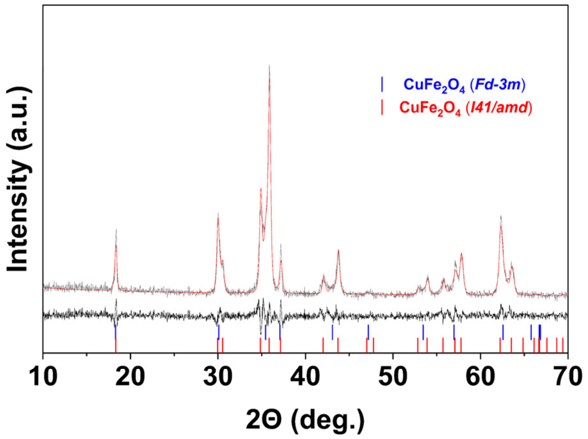

(a)

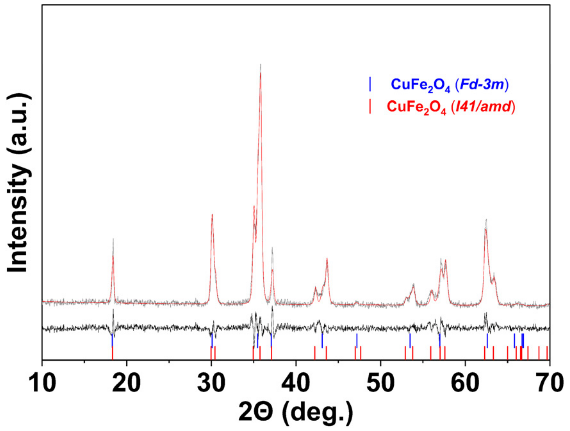

(b)

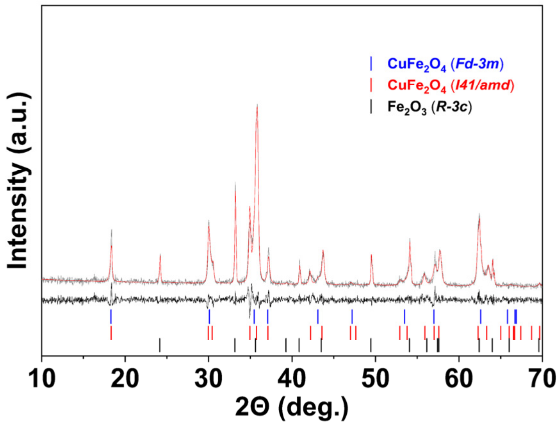

(c)

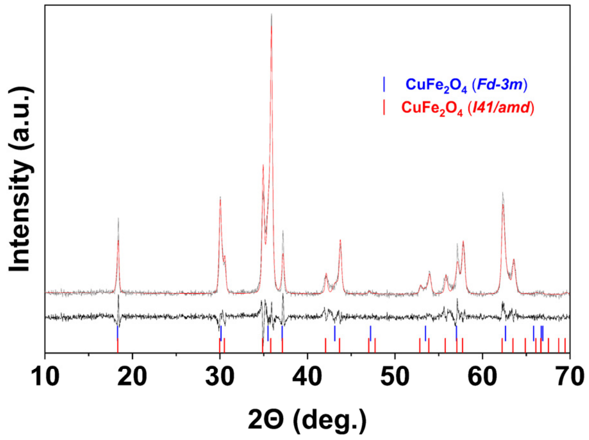

(d)

Figure S1. Rietveld refinement of the XRD pattern of the samples obtained without polysaccharides (a), with dextran-40 (b), with dextran-70 (c), with inulin (d), where the solid curve (grey line) is the observed data and the solid curve (red line) is the best fit, vertical marks indicate the Bragg peaks, and the bottom curve shows the difference between observed and calculated intensities

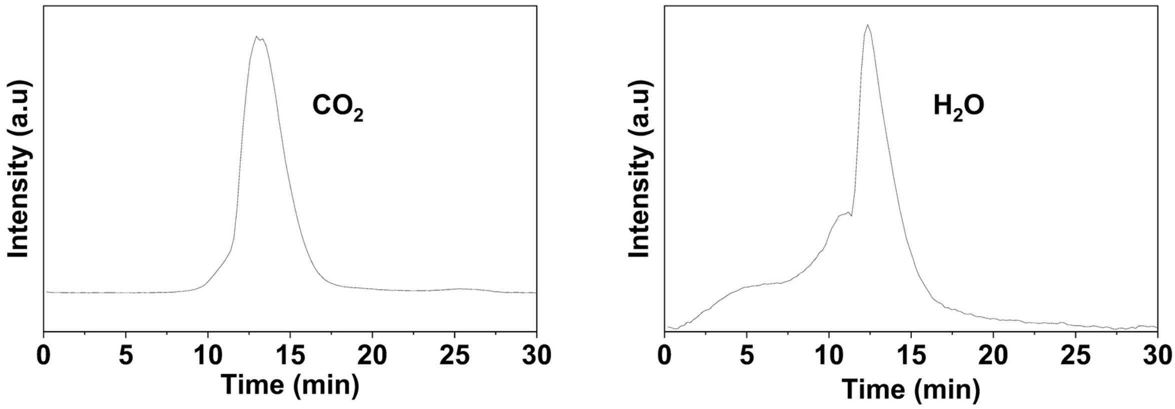

Figure S2. Optical absorption spectra of gases evolved during TGA (thermal decomposition of organic part)

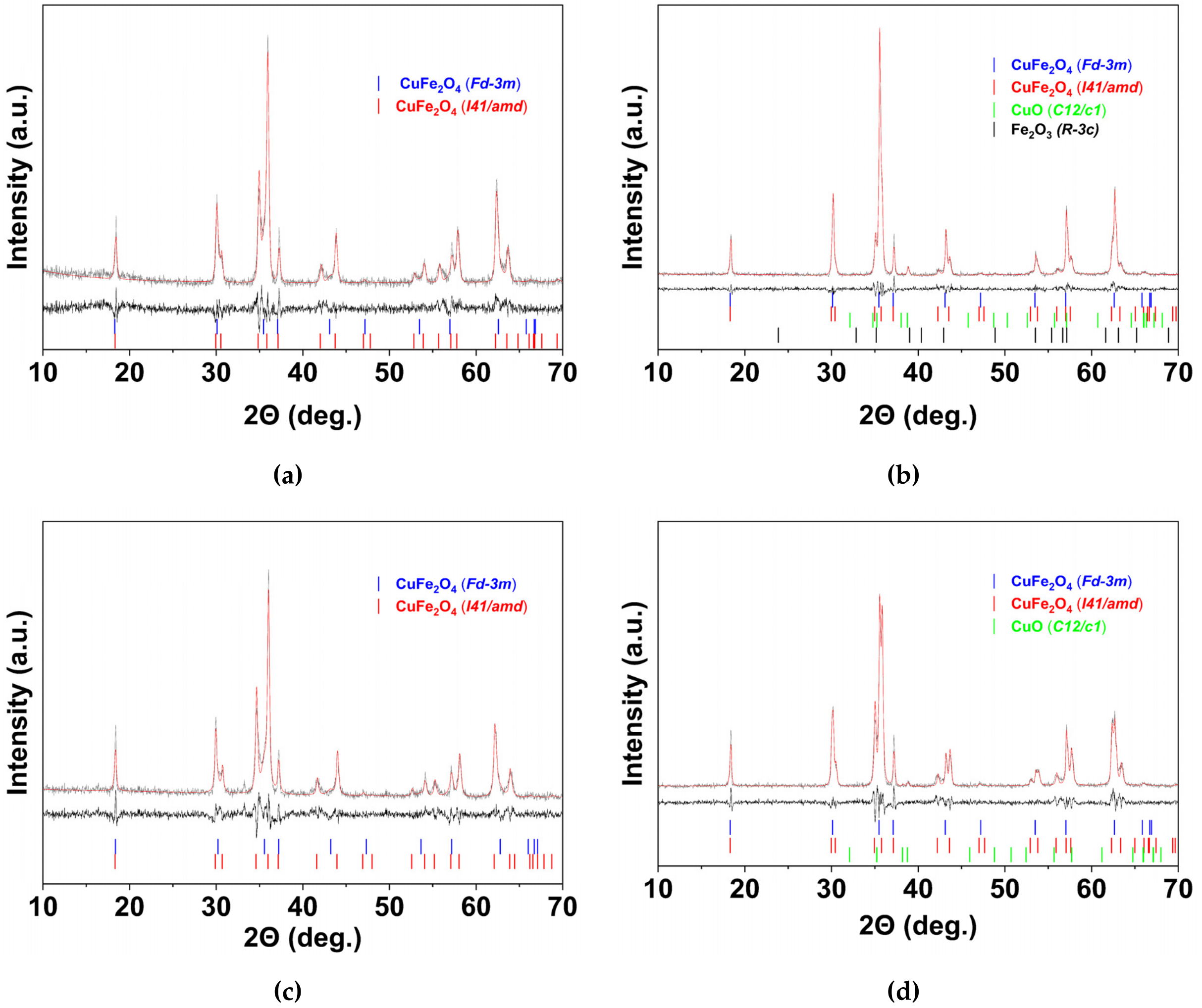

Figure S3. Rietveld refinement of the XRD pattern of samples obtained without polysaccharides (a), with dextran-40 (b), with dextran-70 (c), with inulin (d), where the solid curve (grey line) is the observed data and the solid curve (red line) is the best fit, vertical marks indicate the Bragg peaks, and the bottom curve shows the difference between observed and calculated intensities.

Table S1. Phase composition of samples 1 and 2 (tabl.1) obtained by ion exchange resin precipitation after XRD high-temperature measurements at 900 ° C and after cooling to 25°C

Sample	Polysaccharide	Temperature (°C)	$CuFe_2O_4$ (I41/amd) a	c	ω, wt%	$CuFe_2O_4$ (Fd-3m) a	ω, wt%	CuO ω, wt%	χ2
1	-	900	-	-	-	8.476±0.001	100	-	1.289
		25	5.852±0.001	8.612±0.001	54±3	8.396±0.001	45±3	1.0±0.2	1.129
2	Dextran-40	900	-	-	-	8.484±0.001	100	-	1.109
		25	5.861±0.001	8.601±0.001	51±2	8.394±0.001	47±2	2.0±0.2	1.318

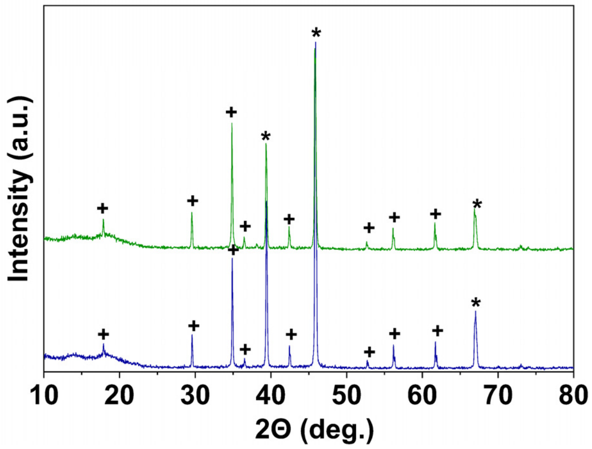

Figure S4. X-ray diffraction patterns of samples 1 and 2 at 900 °C ; top graph – obtained without polysaccharides, bottom graph – obtained with dextran-40. + - cubic $CuFe_2O_4$ (Fd-3m); * - Pt (Platinum)

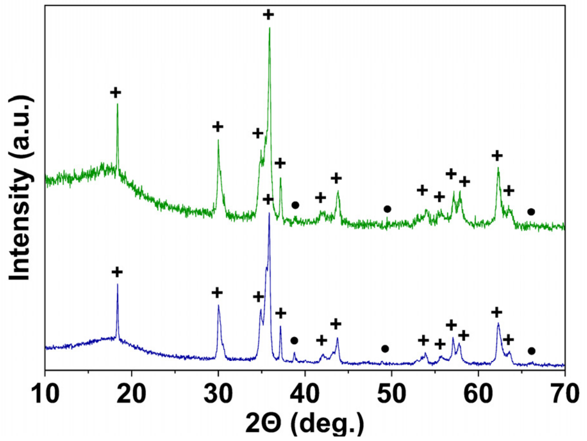

Figure S5. X-ray diffraction patterns of samples 1 and 2 after cooling from 900 °C to 25 °C at a rate of ~30 °C/min; top graph – obtained without polysaccharides, bottom graph – obtained with dextran-40. + - $CuFe_2O_4$; • - CuO

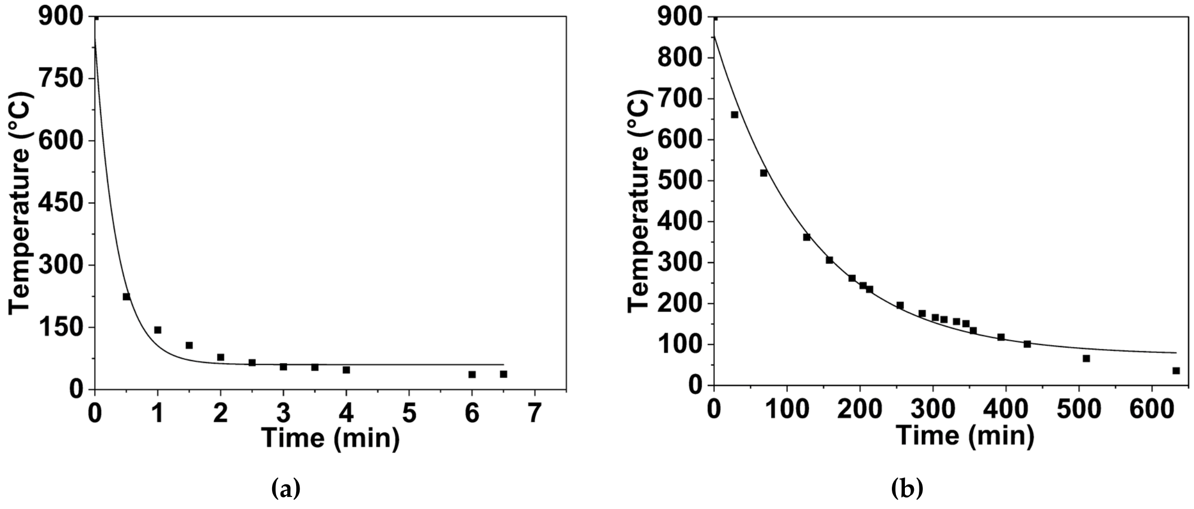

Figure S6. The temperature of the samples as a function of time: a – quenching; b – cooling in the furnace

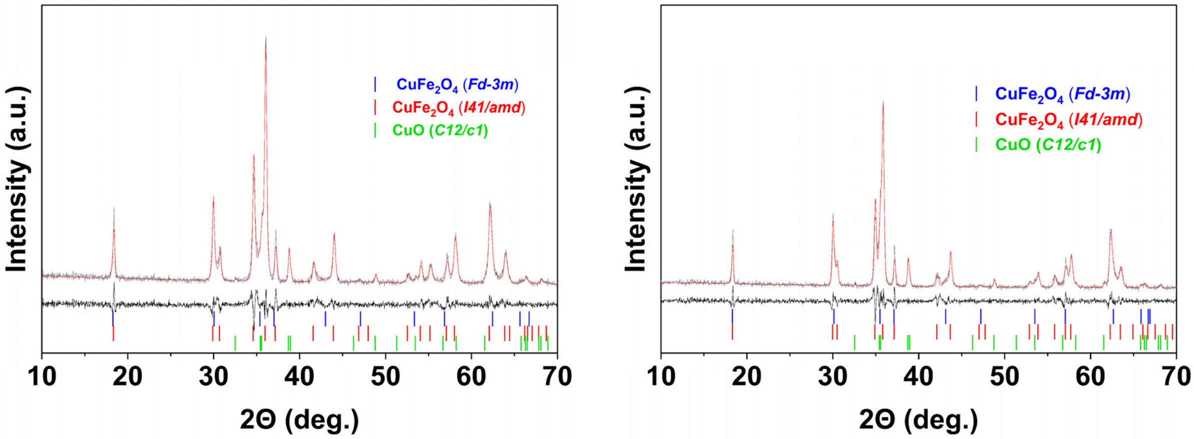

(a) (b)

Figure S7. Rietveld refinement of XRD pattern of samples Cf (a) and Cq (b) where solid curve (grey line) is the observed data and solid curve (red line) is the best fit, vertical marks indicate the Bragg peaks, and the bottom curve shows the difference between observed and calculated intensities.

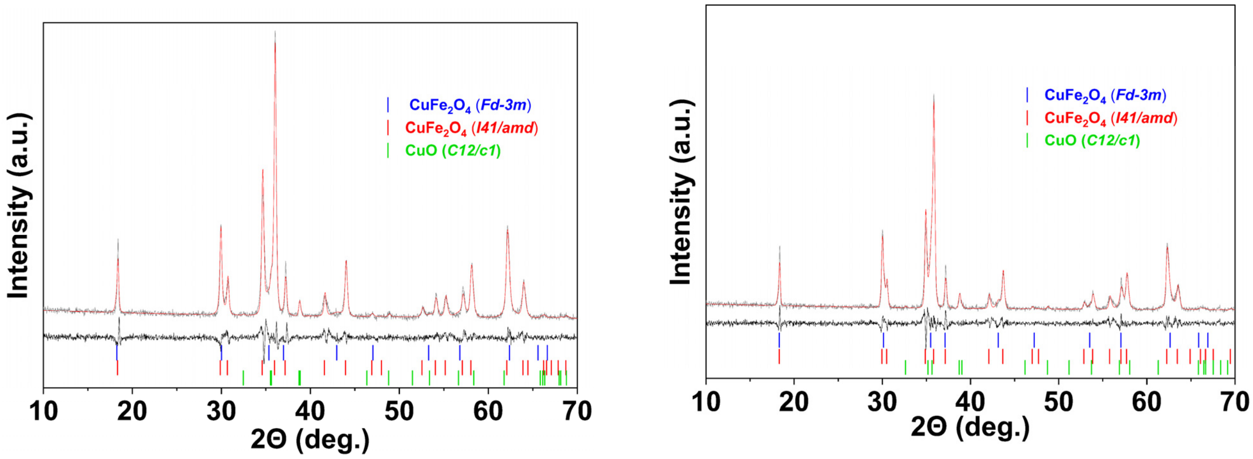

(a) (b)

Figure S8. Rietveld refinement of XRD pattern of samples 1f (a) and 1q (b) where solid curve (grey line) is the observed data and solid curve (red line) is the best fit, vertical marks indicate the Bragg peaks, and the bottom curve shows the difference between observed and calculated intensities.
